# New *Plasmodium vivax* Genomes From the China-Myanmar Border

**DOI:** 10.3389/fmicb.2020.01930

**Published:** 2020-08-11

**Authors:** Awtum M. Brashear, Adam C. Huckaby, Qi Fan, Luke J. Dillard, Yubing Hu, Yuling Li, Yan Zhao, Zenglei Wang, Yaming Cao, Jun Miao, Jennifer L. Guler, Liwang Cui

**Affiliations:** ^1^Department of Internal Medicine, University of South Florida, Tampa, Tampa, FL, United States; ^2^Department of Biology, University of Virginia, Charlottesville, VA, United States; ^3^Dalian Institute of Science and Technology, Dalian, China; ^4^Department of Immunology, College of Basic Medical Science, China Medical University, Shenyang, China; ^5^NHC Key Laboratory of Systems Biology of Pathogens, Institute of Pathogen Biology, Chinese Academy of Medical Sciences and Peking Union Medical College, Beijing, China

**Keywords:** *Plasmodium vivax*, malaria, China-Myanmar border, genome assembly, next-generation sequencing

## Abstract

*Plasmodium vivax* is increasingly the dominant species of malaria in the Greater Mekong Subregion (GMS), which is pursuing regional malaria elimination. *P. vivax* lineages in the GMS are poorly characterized. Currently, *P. vivax* reference genomes are scarce due to difficulties in culturing the parasite and lack of high-quality samples. In addition, *P. vivax* is incredibly diverse, necessitating the procurement of reference genomes from different geographical regions. Here we present four new *P. vivax* draft genomes assembled *de novo* from clinical samples collected in the China-Myanmar border area. We demonstrate comparable length and content to existing genomes, with the majority of structural variation occurring around subtelomeric regions and exported proteins, which we corroborated with detection of copy number variations in these regions. We predicted peptides from all *PIR* gene subfamilies, except for *PIR* D. We confirmed that proteins classically labeled as *PIR* D family members are not identifiable by PIR motifs, and actually bear stronger resemblance to DUF (domain of unknown function) family DUF3671, potentially pointing to a new, closely related gene family. Further, phylogenetic analyses of *MSP7* genes showed high variability within the *MSP7-B* family compared to *MSP7-A* and *-C* families, and the result was comparable to that from whole genome analyses. The new genome assemblies serve as a resource for studying *P. vivax* within the GMS.

## Introduction

The Greater Mekong Subregion (GMS) is problematic in the grand scheme of malaria control due to the repeated emergence of antimalarial resistance in this region (Cui et al., 2012; WHO, 2016; Imwong et al., 2017). The World Health Organization has declared a goal to eliminate malaria in the GMS by 2030 (WHO, 2016). Due to the uneven distribution of malaria along international borders within the GMS, cross-border transmission of malaria from areas with high endemicity to malaria-free areas is a concern (Lo et al., 2015, 2017; Kittichai et al., 2017). Myanmar is one of the six countries within the GMS with the highest malaria burden (Cui et al., 2012; WHO, 2018). The China-Myanmar border is an example of an area wherein cross-border migration is particularly concerning and can result in outbreaks in the less endemic area (Duan et al., 2013; Lo et al., 2015, 2017).

As of 2016, *Plasmodium vivax* was responsible for between 40 and 60% of malaria cases in the GMS countries, and the vast majority of malaria on the China-Myanmar border (WHO, 2018; Geng et al., 2019). *Plasmodium vivax* has a hypnozoite stage which will go dormant for long periods of time before re-emerging to cause relapses. This biological feature makes this parasite resilient to conventional control measures that are designed to treat *Plasmodium falciparum*. Hypnozoites can be treated with primaquine or tafenoquine, but there are contraindications for people with the glucose-6-phosphate dehydrogenase deficiency or for pregnant women and young children (WHO, 2015). Additionally, there are indications that chloroquine resistance in *P. vivax* has emerged in areas of the GMS, which may further create a challenge for treatment (Price et al., 2014). There is no working *in vitro* system to culture *P. vivax* and therefore researchers are increasingly using whole genome studies from field samples in order to answer questions about *P. vivax* evolution (Winter et al., 2015; Hupalo et al., 2016; Pearson et al., 2016; Chen et al., 2017; de Oliveira et al., 2017). However, the lack of samples with high parasitemia has also created barriers to creating necessary genomic resources such as reference genomes.

After the completion of the *P. falciparum* 3D7 genome there was ambition to build a similar genomic reference for *P. vivax* (Carlton, 2003). However, the inability to continuously culture *P. vivax in vitro* poses a barrier to using the same methods (Carlton et al., 2008). The original assembly of the Sal-I strain from El Salvador was highly fragmented consisting of over 2000 extra-chromosomal contigs, and as technology improved, it was found to be missing over 800 genes (Hester et al., 2013). In recent years the focus has been on assembling *P. vivax* genomes based on short reads generated from Illumina sequencing paired with bioinformatic processing through increasingly advanced assembly algorithms. Advancing technology has provided a closer view of the core *P. vivax* genome content while gaining additional insights into the nuances of variability within genomes (Hester et al., 2013; Auburn et al., 2016; Chen et al., 2017). In particular, the assembly of the PvP01 strain from Papua Indonesia revealed an astoundingly large repertoire of subtelomeric multi-gene families (Auburn et al., 2016). It has become clear that there is a high degree of genetic heterogeneity in *P. vivax* parasites (Hupalo et al., 2016). Therefore, researchers have begun to build draft *P. vivax* genomes corresponding to different geographical regions (Menard et al., 2013; Auburn et al., 2016; Chen et al., 2017). Previously, we have shown that malaria parasites from the China-Myanmar border were genetically separated from other parts of the GMS and potentially represent a unique genetic profile resulting from the clonal expansion of a few parasite strains during an outbreak (Brashear et al., 2020). Unfortunately, the closest *de novo* assembly and analysis performed previously from this region was heavily fragmented, consisting of over 8,000 different scaffolds and identifying a small portion of subtelomeric *pir* genes (Chen et al., 2017). Thus, we generated representative *P. vivax* genomes from this region to provide additional insights into the evolution of this parasite population. Here, we performed *de novo* genome assembly on whole genome sequencing (WGS) data from four *P. vivax* field isolates from the China-Myanmar border.

## Materials and Methods

### Ethics Statement and Sample Collection

This research was approved by local Bureau of Health of Kachin and the Institutional Review Board of Pennsylvania State University IRB #34319. Patients with acute *P. vivax* malaria presented at the hospital and clinics of Laiza town, Kachin State, Myanmar and Nabang Township, Yunnan Province, China were recruited after obtaining written informed consent. Malaria diagnosis was performed by microscopic examination of Giemsa-stained blood smears. Five milliliters of venous blood were drawn by venipuncture from each patient and filtered to remove human leukocytes as previously described (Li et al., 2017). Parasites were released after lysis of the red blood cells (RBCs) with saponin, pelleted by centrifugation, and stored at −80°C until DNA extraction.

### DNA Library Preparation and Illumina Sequencing

Parasite DNA was extracted from the parasite pellets using the QIAamp DNA Mini Kit (Qiagen, Hilde, Germany) and air-dried for storage. DNA from each sample was resuspended in 20 μL of water and DNA concentration was measured on a Qubit Fluorometer. DNA libraries were prepared using the TruSeq Nano DNA Library Prep Kit with up to 100 ng of DNA, which was sheared to create 350 nt inserts. DNA libraries were sequenced on the HiSeq 2500 in rapid mode to create 150 bp paired-end reads. DNA reads were trimmed for quality, removing areas with a Phred score under 20 using trimmomatic (Bolger et al., 2014).

### Selection of Isolates for Genome Assembly

Reads were aligned to the reference PvP01 genome using BWA MEM (Li and Durbin, 2010). Samtools v1.3 was used to characterize average coverage over the reference genome, and those with an average coverage under 10 × were excluded (Li et al., 2009). To account for multiple infections, we estimated multiplicity using estMOI (Assefa et al., 2014). For confirmation, we cross referenced these results with visual analysis of the highly polymorphic merozoite surface protein 1 (*MSP1*) gene using integrative genomics viewer (IGV) (Thorvaldsdottir et al., 2013). We excluded samples for which multiplicity was higher than 1. For samples that passed these filters, *P. vivax* genomes were assembled using Velvet assembler at k-mer of 31, 51, 71 and 91 (Zerbino and Birney, 2008). Assembly quality statistics were assessed via QUAST (Gurevich et al., 2013) with manual review focusing on N50, genome length, and the proportion of misassembled contigs. From these statistics we chose the best-performing k-mer for each sample; if two assemblies seemed comparable, we chose the larger of the two in order to better resolve repetitive regions. To improve the assembly and reduce the number of contigs, each sample’s best assembly was scaffolded with SSPACE (Boetzer et al., 2011), had gaps filled with gapfiller (Nadalin et al., 2012), and was error-corrected with Pilon (Walker et al., 2014).

### Genome Refinement

The four samples performed the best in terms of N50 and contiguity were pseudo-contiguated with ABACAS2 (Assefa et al., 2009), gap-filled with IMAGE (Tsai et al., 2010) and corrected with Pilon. Assembled and corrected genomes were uploaded to the Companion webserver for multi-faceted gene annotation (Steinbiss et al., 2016). PvP01, obtained from a patient from Papua Indonesia, was used as the reference genome (Auburn et al., 2016), and annotations were performed with reference protein evidence as well as RATT annotation transfer at the strain level. All other options were left at default options, including pseudo-contiguation with a 500 bp match length and 85% similarity. For consisteny between genome annotation, assembled genome statistics for new assemblies including size, N50 and #Ns/kbp were calculated using post-companion genomes using QUAST, omitting sequences less than 500 bp. Because companion pseudo-contiguation bins extrachromosomal scaffolds together and to minimize chimeric extrachromosomal contigs, we separated each stretch of nucleotides separated by 100 or more N characters.

### Gene Characterization

GFF files were downloaded for each sample from the companion annotation and used for further gene analysis. Gene lists and predicted protein sequences were extracted from the genome of each field sample and the PvP01 reference genome. Genes were either identified by the PvP01 orthologs or their Pfam identifiers when possible. Additionally, genes were highlighted along each chromosome using Circos (Krzywinski et al., 2009). In addition, Circos plots visualized the depth of supporting reads based on BWA alignment and segments without high identity to P01 which were found using blat comparisons using a 98% identity threshold and the resulting UCSC chain files were used to identify conserved regions and the remaining regions were plotted (Kent, 2002).

Gene predictions from each assembly were input into OrthoVenn2 (Xu et al., 2019) to detect overall ortholog conservation between assemblies. For the MCL analysis, we used an e-value of 1 × 10^–5^ and an inflation factor of 1. The top four with the lowest number of gaps were used to characterize the gene content of each assembly. Reference genes were annotated using PlasmoDB^1^, while new genes were annotated using a combination of companion annotations and BLAST search of PlasmoDB and GenBank.

A combination of PFAM-based and custom Profile Hidden Markov Models (HMMs) were used to detect the presence of gene families. HMMer was then used to scan the predicted genes of each genome assembly (Eddy, 2011). For confirmation, HMM searches were performed on both the Sal-I and PvP01 reference genomes. Additional protein family profiles were constructed for each of 10 *pir* gene subfamilies based on previously classified PIR proteins (Apweiler et al., 2004). Skylign was used for visualization of HMM profile motifs, except for the DUF3671 group which was selected from the PFAM database (Wheeler et al., 2014).

### Phylogeny and Genetic Diversity

Reads for PvP01 and each of the four samples were aligned to the PvP01 reference using BWA-MEM. We then created a maximum likelihood tree with RAXML using the GTR model with Lewis ascertainment correction. In all cases bootstrapping was set to test for convergence (bootstopping) with a maximum of 1000 iterations, and in the case of the whole genome phylogeny, convergence was reached after 50 bootstrapping replicates. The phylogenetic trees were visualized using ggtree (Yu et al., 2017). Similarly, phylogenetic relationships of *pir* and *msp7* genes in four newly assembled *P. vivax* genomes were evaluated using the maximum likelihood algorithm in RAxML-NG with a JTT model. Additionally, we performed identical variant calling against the PvP01 reference for the four assembled samples and analyzed their nucleotide diversity in 1 kb sliding windows using vcftools (Danecek et al., 2011).

### Copy Number Variation (CNV) Detection

Copy number variation for four new genome assemblies were identified as previously with several modifications (Huckaby et al., 2019). Briefly, BWA-MEM was utilized to align reads with default settings to the PvP01 reference genome. Breakpoints of the CNVs were identified using the Speedseq pipeline which includes CNVnator for read-depth analysis and LUMPY for discordant and split-reads analysis. Additionally, a final Speedseq step included a Bayesian likelihood analysis for genotyping of structural variants, which analyzes the ratio and quantity of discordant or split-reads to concordant read-pairs and reads. The settings were modified to require a minimum of two supporting discordant read pairs or split-reads for LUMPY and utilize 1000 bp windows for CNVnator read-depth analysis and the option for genotyping by SVTyper within Speedseq (Chiang et al., 2015).

To generate a final high confidence CNV set, several exclusion criteria were applied. First, CNVs were excluded if their lengths were <2000 bp or >100,000 bp. CNVnator with 1000 bp windows cannot call CNVs <2000 bp and CNVs > 100,000 bp are frequently false positives. Next, CNVs with Speedseq quality scores of <0 were disregarded in order to exclude randomly mapped discordant read-pairs and split-reads (due to sequence homology at multiple locations). CNVs on PvP01 contigs were also excluded as the confidence in their assembly is less than the full PvP01 chromosomes. For final high-confidence CNV calls, any CNVs without > 50% reciprocal overlap of both LUMPY and CNVnator calls were excluded. The boundaries of the resulting high-quality CNVs were reported using the LUMPY locations as discordant read-pairs and split-reads give greater resolution for breakpoints. When multiple LUMPY calls were found to overlap in a single sample, and these resulted in a similar length to a CNVnator call (i.e., CNVs on chromosome 9), boundaries were reported as the outer limits of the LUMPY CNV calls.

## Results

### Genome Sequencing and Assembly

We obtained 23 *P. vivax* field samples from the China-Myanmar border: four from the Nabang town on the China side and 19 from Laiza town on the Myanmar side. The number of reads obtained for each sample ranged from 7,252,842 to 9,529,810 with the median being 8,387,821. After removing poor-quality reads and reads matching the human genome, the remaining number of reads ranged from 376,875 (4.3% of total reads) to 6,663,732 (77.9%) with the median being 3,278,840 (36.7%). Alignment to the PvP01 reference genome revealed that the average depth per nucleotide for each sample ranged from 2.6 to 61.7 with the median average depth being 29.1 (Supplementary Figure S1A).

**FIGURE 1:**
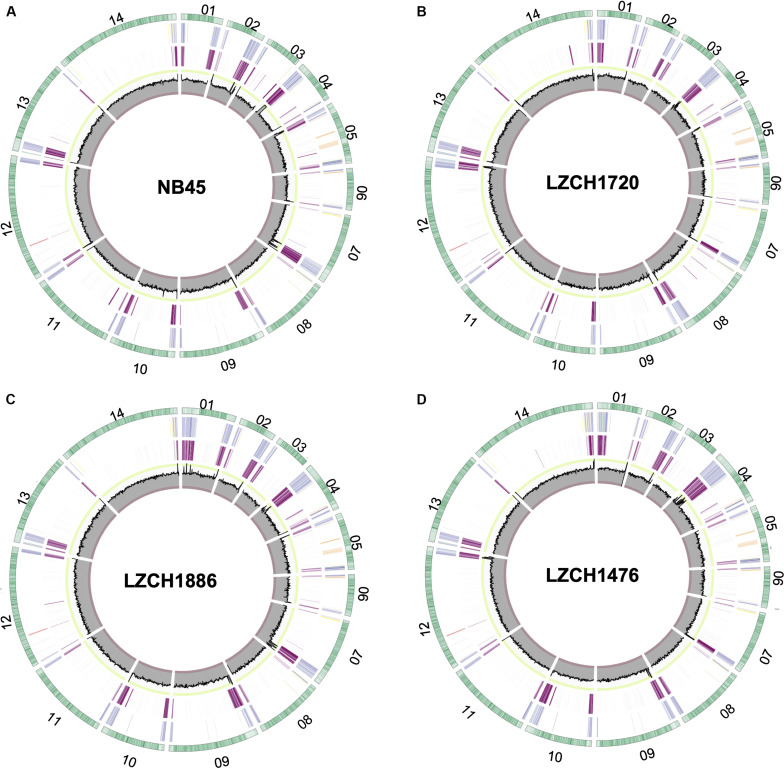
Circos plots for the four *P. vivax* genome assemblies **(A)** NB45, **(B)** LZCH1720, **(C)** LZCH1886, and **(D)** LZCH1476. The outermost green circle represents genes on each chromosome. The second ring colors in select gene families wherein PIR genes are blue, DUF3671 genes are teal, MSP7 genes are red, RBPs are yellow, PHIST genes are orange, and STP1 genes are green. The 3rd ring highlights regions which do not share 98% identity with PvP01. The innermost circle represents coverage of reads for the assembly wherein the minimum is 0, the maximum is 100. To provide context, the interval between 90 and 100 is shown in green, and the interval between 0 and 10 is shown in red.

We performed *de novo* assembly on 14 field samples of *P. vivax* infections with the highest coverage, and the assembled genomes had a median N50 of 38,858.5 and a median number of contigs of 2143 (Supplementary Figures S1B–D). Four samples with the highest N50 values prior to correction, and confirmed as monoclonal using three separate methods (Supplementary Figure S2), were selected for detailed characterization. These were pseudo-contiguated and corrected for downstream analysis. The median depth of these four samples was 58.87 and coverage remained consistently high throughout the chromosomes (Figure 1). After processing, the four *P. vivax* genomes had a median N50 of 1,781,807, a median number of scaffolds of 83.5, and average genome size of 29.86 Mbp, slightly larger than the PvP01 reference (Table 1). After excluding the extra-chromosomal contigs, apicoplast and mitochondrial genomes, on average, 92.80% of chromosomes of the four genomes aligned to PvP01.

**TABLE 1:** Genome statistics for each of the four *P. vivax* field samples and two reference genomes.

	Sal-I	P01	NB45	LZCH1476	LZCH1886	LZCH1720
Reads sequenced	NA	NA	15343644	12553940	12799214	12929808
Average depth			58.51	58.04	59.23	60.07
Total genome length (Mbp)	27.01	29.05	30.05	29.88	29.53	29.84
Chromosome length (Mbp)	22.62	24.21	25.00	25.54	25.61	24.93
Ns/100 kbp	199.53	517.08	1034	1327.3	1365.97	1337.33
Number of scaffolds	2748	242	139	73	65	94
N50 (bp)	1678596	1761288	1743222	1783114	1780500	1920463
% Alignment to P01	79.31%	99.48%	81.43%	81.50%	81.06%	81.36%

### Genetic Diversity

Our previous analyses of single nucleotide polymorphisms (SNPs) from samples collected at the China-Myanmar border revealed that three samples (LZCH1720, LZCH1476, and LZCH1886) were closely related (Brashear et al., 2020). To estimate diversity of the four genomes used for assembly, we characterized the degree of diversity within the primary chromosomes. We identified 110,635 high-quality bi-allelic SNPs compared to the PvP01 reference. Sliding window analysis of nucleotide diversity revealed high diversity at the subtelomeric regions and the median nucleotide diversity among all windows was 4.29 × 10^–4^ (Supplementary Figure S3).

### Genome Annotations

Genes were predicted for each of the four newly assembled *P. vivax* genomes. On average 6721 genes were predicted as compared to 6741 genes for the PvP01 genome. To validate the post-assembly pipeline, the PvP01 genomes were put through an identical post-processing and gene finding pipeline. The results showed 99.85% identity for the PvP01 gene annotation within the core chromosomes. This high degree of consistency allowed us to use polypeptides from the published PvP01 annotation as the reference for comparison with the new assemblies. Meanwhile, new assemblies consistently had between 92.5 and 93% of their chromosomes aligned to those of the PvP01, whereas the Sal-I assembly only had 90.75% of its chromosomes aligned to those of the PvP01 reference. In each of the four assemblies, areas of low conservation were concentrated around the chromosome ends (Figure 1).

**FIGURE 2:**
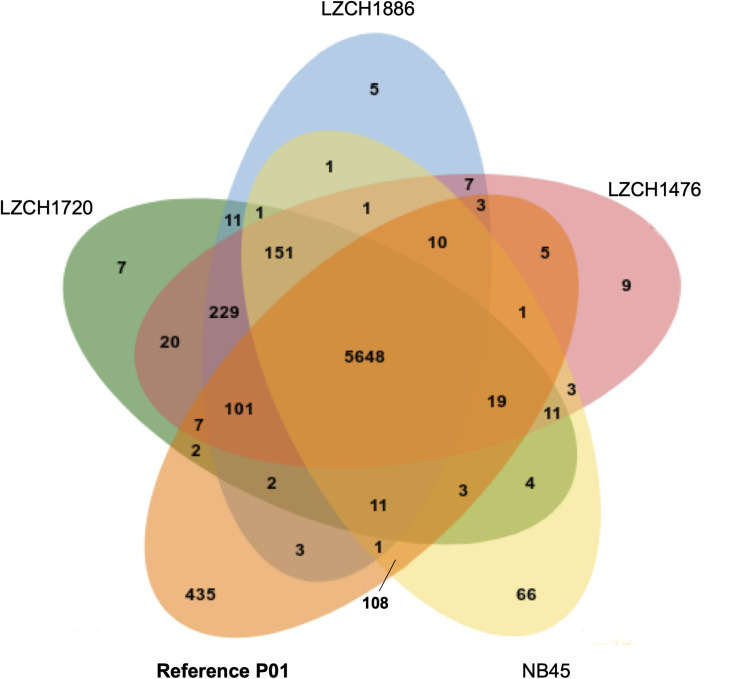
Ortholog presence in each of the four China-Myanmar border *P. vivax* isolates compared to reference PvP01 genome. Each color represents all ortholog groups present in each of 5 different isolates, with the reference P01 assembly being shown in orange.

The predicted genes for the four new assemblies as well as PvP01 and Sal-I references were assigned into orthologous groups, which resulted in 6916 orthology clusters including 4843 single-copy gene clusters (Figure 2). The four genomes had an average of 6598 predicted peptides for comparison to the Sal-I genome (5389 annotations) and PvP01 genome (6677 annotations) (Table 2). There were 514 coding genes which were present in reference genomes but missing in all four new assemblies (Figure 2). These genes primarily consisted of multi-gene family members including *pir* (336), *phist* (4), *stp1* (2), and *msp3* (3). Additionally, many of the missing genes encode proteins with unknown functions such as exported proteins with unknown functions (138), conserved *Plasmodium* proteins with unknown function (9) and hypothetical proteins (9). Additional genes annotated in the reference genomes but not found in the new assemblies include 4 apicoplast ribosomal proteins, 2 ribosomal proteins, 1 reticulocyte binding protein 1a, 1 putative helicase, 1 carbonic anhydrase pseudogene, 1 PPPDE peptidase, 1 palmitoyltransferase DHHC9, 1 porphobilinogen synthase, and 1 KS1 protein precursor.

**TABLE 2:** Gene content of each of 4 genomes chosen for gene analysis compared to references Sal-I and PvP01.

Annotation	Sal-I	P01*	LZCH1720	LZCH1886	NB45	LZCH1476
Encoded peptides	5389	6677	6606	6530	6655	6601
Genes without PvP01 orthologs	60	34*	712	543	869	548
PIR	387	1181	1157	1111	1204	1164
ETRAMP	10	10	10	10	10	10
PHIST	79	81	77	76	77	76
STP1*	13	10	8	10	16	9
RBP*	10	10	9	9	9	9
MSP7	11	11	11	11	11	11
Tryptophan-rich antigens	36	40	40	40	40	40

*P01 numbers reflect an identical annotation pipeline imposed on the P01 genome sequence in order to control for differences in methodology and are therefore not identical to existing annotations.

### Newly Identified Genes

There were 151 new ortholog clusters which contained genes without direct orthologs in PvP01; 120 of these contained just one gene from each of the four assemblies. Of the 120 new clusters, 65 were characterized as *pir* genes, 22 were proteins of the unknown functions and/or had the domain of unknown function, DUF3671, and 33 were hypothetical proteins with no known pfam domain match. The 31 gene clusters which had more than one ortholog in at least one genome assembly accounted for 44 new genes in NB45, 45 new genes in LZCH1720, and 46 new genes in both LZCH1476 and LZCH1886. Of the 31 genes, 19 were *pir* genes, six were genes of unknown function, and six were hypothetical proteins with no known pfam domain match.

### Multigene Families

The majority of differences noted were within multigene family members, and areas of low conservation seemed to coincide with subtelomeric regions while the core genomes were relatively conserved (Figure 1). This creates the possibility that many new or missing genes may be mutually present as part of highly variable gene families, and are therefore very divergent from their P01 orthologs. To verify the overall genome assembly results of the multigene families, we extracted all members for the *pir*, *phist*, *stp1*, *rbp*, *msp7*, and *etramp* gene families from the four new *P. vivax* assemblies and the reference genomes Sal-I and PvP01 (Table 2). The *pir* gene family repertoire ranged from 1111 to 1204 in the four new *P. vivax* genomes as compared to 1181 in the PvP01 genome and 387 in the Sal-I genome. Three new assemblies had 8–10 *stp1* family members similar to PvP01 with 10 members, whereas the NB45 genome had 16. Each of the four newly assembled *P. vivax* genomes had 76 or 77 *phist* family members (compared to 79 and 81 in Sal-I and PvP01, respectively), 9 *rbp* genes (compared to 10 in both references), and 40 *tryptophan-rich antigen* genes (compared to 36 and 40 in Sal-I and PvP01, respectively). Each of the new assemblies had the same number of genes as in Sal-I and PvP01 for *msp7* (11 members) and *etramp* (10 members). It is noteworthy that due to high levels of diversity, some of the gene family members were not clustered via orthology with genes in PvP01. For example, of the 548 genes without a PvP01 ortholog in LZCH1476, 356 were *pir* genes, 77 had similarity to DUF3671 (a gene with an unknown function), and one was an *stp1* gene. Overall, of the genes within new multi-gene orthology clusters, the majority (between 30 and 32 genes in each sample) had domain similarity to *pir* proteins, and between 7 and 11 for each assembly had DUF3671 domain structures. Segments containing many of gene family members, particularly *pir* and *msp7* gene family members, coincided with low conservation regions (Figure 1).

### Pir Subfamily Identification

**FIGURE 3:**
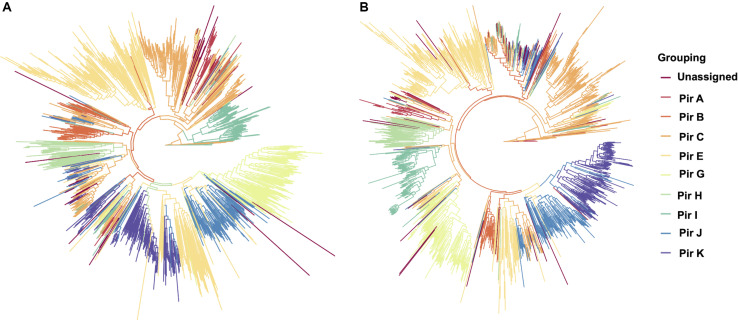
Maximum-likelihood PIR family structure within two assembled genomes **(A)** NB45 and **(B)** LZCH1720. Each color represents a separate subfamily as denoted by the color key on the right.

To establish that missing orthology within a subset of *pir* genes was not an outcome of missing gene family members but rather due to more stringent classification methods, we assigned the extracted *pir* genes to *pir* gene subfamilies as previously defined (Lopez et al., 2013). In each of the four newly assembled samples, nearly all *pir* genes were mapped to at least one *PIR* subfamily (Table 3). In the LZCH1886, LZCH1476, LZCH1720, and NB45 assemblies, there were 35, 38, 38 and 33 predicted PIR proteins, respectively, unassigned to PIR subfamilies. Because our reported estimates of *pir* genes is similar to previously reported (Auburn et al., 2016), and in order to establish subfamily diversity, we examined the composition of *pir* subfamilies. We created a maximum likelihood tree for predicted PIR proteins in two selected samples. We found clustering of samples from the same gene subfamilies, with the largest gene subfamily consistently being PIR E, closely followed by PIR C. Interestingly, while the majority of unassigned PIR proteins formed a clade with the PIR C subfamily in the NB45 genome assembly, they formed a more distinct clade in the LZCH1720 assembly (Figure 3).

**TABLE 3:** Count of PIR protein subfamilies within PIR orthologous genes.

Subfamilies	LZCH1720	LZCH1886	NB45	LZCH1476
PIR A	32	32	37	32
PIR B	53	47	51	53
PIR C	227	217	225	227
PIR D	0	0	0	0
PIR E	231	225	256	238
PIR G	117	109	130	114
PIR H	51	47	47	51
PIR I	124	122	128	124
PIR J	138	133	143	138
PIR K	154	144	154	154
Unassigned	38	35	33	38

The subfamily D had no members within the PIR families of any of the newly assembled *P. vivax* genomes (Table 3). To establish what may be causing a depletion of this gene family, we ran an HMM profile scan against all proteins in the genome using previously identified PIR D family members and found that there were 270, 280, 260 and 272 proteins within the entire predicted protein database of the newly assembled *P. vivax* genomes LZCH1720, NB45, LZCH1886, and LZCH1476, respectively. Each of the identified proteins had high similarity scores to the profile of the constructed PIR D subfamily. Interestingly, despite these similarities to the PIR D subfamily, most of the proteins had higher similarity to the DUF3671 domain. Specifically, PIR D subfamily members accounted for 253/254 (99.6%), 237/238 (99.5%), 247/248 (99.6%), and 259/260 (99.6%) of DUF3671 proteins in LZCH1476, LZCH1886, LZCH1720 and NB45, respectively.

To determine if DUF3671 domain characterizes a separate gene family which overlaps with the previously designated PIR D gene subfamily, we performed analysis of both gene domain homologies. Within the Pfam database there are 489 sequences which contain the DUF3671 domain, all of which belong to a species of non-falciparum *Plasmodium*; 72% from *P. malariae*. We also compared motifs between the DUF3671 profile available from Pfam, and the constructed HMM profile based on suggested PIR D family members, finding that two of the most conserved profile components from the PIR D profile were similar to two of the most conserved profile components from the DUF3671 profile (Supplementary Figure S4). Meanwhile, neither of the architecture components were present in the overall PIR protein profile, and none of the major components of the PIR protein profile were present in the profile of PIR D.

### MSP7 Diversity

**FIGURE 4:**
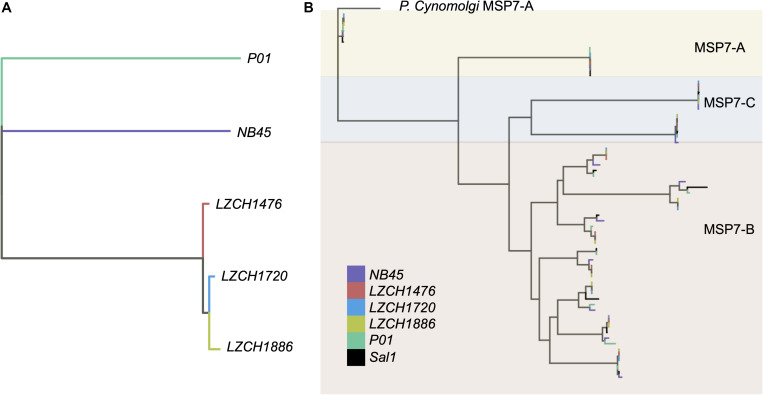
Genetic diversity within *P. vivax* assemblies. **(A)** Genetic diversity within the entire genome of the four assemblies compared to PvP01 based on SNPs from alignment to Sal-I. **(B)** Diversity within *msp7* genes between different assemblies, Sal-I and PvP01. *P. cynomolgi* MSP7-A was used as an outgroup. Colors: Black– P01, Blue – Sal-I, Orange—NB45, Red—LZCH1720, Cyan—LZCH1476, Purple—LZCH1886.

Genes from the *msp7* family were consistently represented with 11 genes in each assembly (Table 2). *Msp7* genes tend to be variable and are therefore not well characterized by SNP data. Our assemblies all showed low conservation to PvP01 around the *msp7* locus (Figure 1), so we characterized diversity within the *msp7* gene family within the newly assembled genomes (Figure 4, Supplementary Figure S5). To establish general relatedness of the parasite for comparison, we first constructed a phylogenetic tree based on SNPs from the four new assemblies (Figure 4A, Supplementary Figure S5B), which consistently revealed the closer relationship among three new genome assemblies. Phylogenetic tree of predicted MSP7 proteins showed that the MSP7-C and MSP7-A orthologs, consisting of two protein clades each, were highly related between these assemblies (Figure 4B, Supplementary Figure S5), highlighting evolutionary conservation of these genes. MSP7-B subfamilies, consisting of the remaining 7 proteins, were much more diverse. Within each protein cluster, the PvP01 and Sal-I MSP7-B protein formed a separate clade from the MSP7-B proteins from China-Myanmar border assemblies (Figure 4B, Supplementary Figure S5B). In two instances, however, NB45 appeared to form a clade with the PvP01 and Sal-I references rather than the other China-Myanmar border samples (Figure 4B, Supplementary Figure S5B).

### Copy Number Variations (CNVs)

**FIGURE 5:**
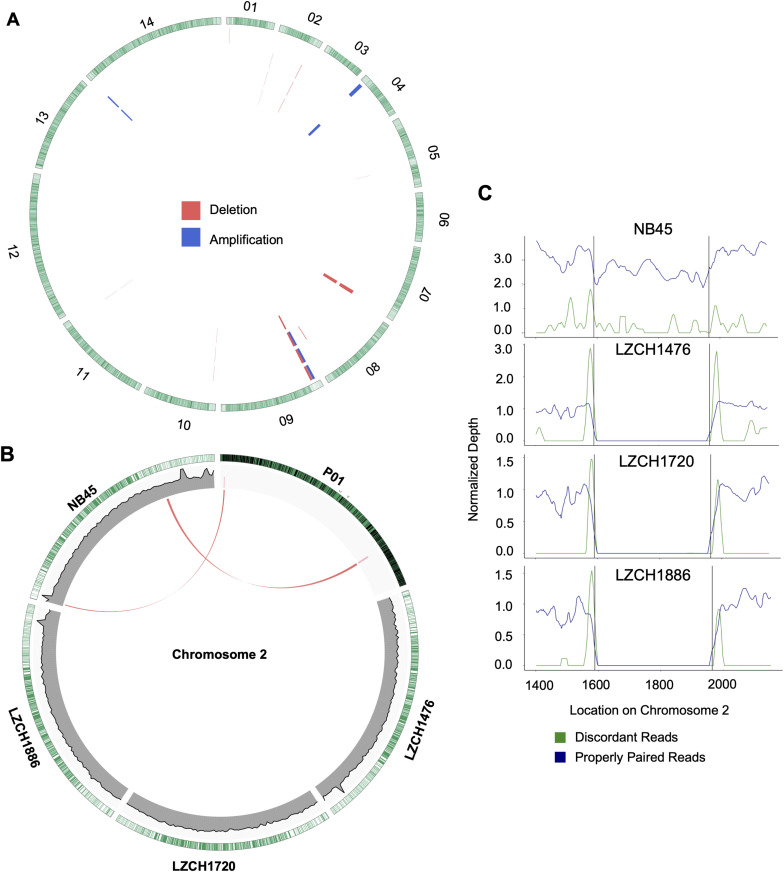
Structural variation within assembled genomes. **(A)** Locations of all predicted copy number variations. Each circle represents a different sample in this order: NB45, LZCH1476, LZCH1720, and LZCH1886. Pink wedges represent predicted deletions while blue wedges represent predicted amplifications. Outer ring shows gene density wherein green highlights are individual genes. **(B)** CNVs on chromosome 2 with wedges representing their location within PvP01 and lines connecting them to homologous regions on respective assemblies when applicable. Coverage support plots were included for the four new assemblies in the second ring. **(C)** Normalized depth for both discordant and properly paired reads around a chromosome 2 deletion predicted for 3 of the 4 samples. Black lines denote the predicted deletion boundary.

Copy Number Variations in the new genome assemblies were scanned using different algorithms. After applying several criteria to identify high-confidence CNVs, we identified a final set of five amplifications (averaging 46 kb) and 11 deletions (averaging 27 kb) in the four new assemblies. All of the amplifications were supported by more than one sample and were located in telomeric or subtelomeric regions (Table 4, Figure 5A). A set of three amplifications found in LZCH1720 and NB45 on chromosome 9 appeared to exhibit complex breakpoints as two of the CNVs that were called added up to the largest CNV found at that location (Table 4). Of the deletions, five were identified in individual samples and the other six were present in two or three samples (Table 4, Figure 5). A similar situation was found on chromosome 9 with multiple deletions found just upstream of the amplification loci that were previously mentioned (Table 4, Figure 5). This locus was found to be variable in all four assemblies, with LZCH1476 and LZCH1720 having the same deletion (Table 4, Figure 5A).

**TABLE 4:** Copy Number Variations detected within the 4 isolates compared to PvP01.

Supporting Samples	Type	Chr.	Start	End	Length
LZCH1886, NB45	Amplification	4	38123	124716	86593
LZCH1476, LZCH1720, NB45	Amplification	9	79721	135397	55676
LZCH1720, NB45	Amplification	9	79721	94710	14989
LZCH1720, NB45	Amplification	9	94119	135206	41087
LZCH1476, LZCH1720	Amplification	14	82	35455	35373
NB45	Deletion	1	58587	62858	4271
LZCH1476, LZCH1720, LZCH1886	Deletion	2	15890	19643	3753
LZCH1476, LZCH1720, LZCH1886	Deletion	2	776331	785302	8971
LZCH1720	Deletion	5	1010209	1013244	3035
LZCH1720, LZCH1886	Deletion	7	1531179	1630181	99002
LZCH1720	Deletion	8	1647230	1662256	15026
LZCH1476, LZCH1720	Deletion	9	135520	190955	55435
LZCH1886	Deletion	9	135520	182436	46916
NB45	Deletion	9	141424	191395	49971
LZCH1476, LZCH1720, LZCH1886	Deletion	10	91532	96146	4614
LZCH1720, LZCH1886	Deletion	11	2054089	2057879	3790

*Chr., chromosome.*

Based on current annotations for the PvP01 genome, the majority of genes included in the CNV amplifications were *pir* family members which matches results from the assemblies. However, the amplifications on chromosomes 4 and 14 also included putative exported proteins with unknown functions (Supplementary File 1). The genes involved in deletions were similar to the amplifications with the majority composed of *pir* family members or exported proteins with unknown functions (Supplementary File 1). The complex amplifications and deletions on chromosome 9 included hypothetical proteins.

Genes within the predicted CNVs in the genome assemblies involve many hypervariable gene families. Despite lower resolution of the hyper-variable regions within the newly assembled genomes, direct comparisons to the assembled genome in some cases further corroborated the detection of CNVs. Within chromosome 2, for instance, deletions were predicted on either end of the chromosome for LZCH1720, LZCH1886 and LZCH1476, but not in NB45 (Figure 5, Table 4). Accordingly, while NB45 had regions homologous to the deleted region within its assembled genome, the other three assemblies were missing this section (Figure 5B). Visualization of one of the two regions, between 15890 and 19643 further supported a reduction in properly paired read coverage and increases of surrounding discordant reads for the predicted region for all assemblies except for NB45.

## Discussion

To date, no gapless *P. vivax* genome has been completed, and diversity within *P. vivax* populations results in genomic differences for populations from different geographic areas. The China-Myanmar border, for instance, represents a unique region of malaria endemicity, which would benefit from the availability of high-quality *P. vivax* genome sequence collection from this area. Here we present four draft genomes with similar contiguity to the published reference PvP01 and performed detailed gene annotations. We have demonstrated that their genomic contents are consistent with expectations for the core *P. vivax* genomes. However, some genes, particularly those from antigenic gene families, are highly diverse and frequently not directly orthologous to their counterparts in the current reference genomes.

The *P. vivax* genome assemblies described here are of comparable or higher quality to the existing references. In terms of length and contiguity they parallel the genome assemblies from Papua Indonesia, China and Thailand, which ranged between 28.9 and 30.2 Mbp with 230–529 scaffolds, compared to our samples which covered between 28.5 and 30.1 Mbp within 65–139 scaffolds (Auburn et al., 2016; Table 1). Between 92.5% and 93% of the 14 chromosomes, representing the core nuclear genome, from each assembly aligned to the reference PvP01. Missing bases on the genome were common, ranging from 1034 to 1365.97 Ns/Kbp compared to 517 in the reference. However, we were still able to capture the majority of annotated genes, comparable to or even in excess of the PvP01 reference (Table 2). Only six genes missing from our assemblies were not a member of a multi-gene family. It is worth noting that PvP01 is the most contiguous and highest-coverage assembly used currently, with other assemblies being much more fragmented. It is noteworthy that both the current gold-standard reference and the new assemblies presented here contain a number of ambiguous sequences and extra-chromosomal contigs, which are intrinsic to the short-read sequencing technology. The next major improvements in genome contiguity will likely occur when *P. vivax* genomic DNA can be obtained in the quantities required for long-read sequencing.

Our data indicate few large-scale variations in the core genome and high variability in multi-gene families and subtelomeric regions, also supported by high nucleotide diversity and occurrence of CNVs toward chromosomal ends (Supplementary Figure S1, Figure 5). Subtelomeric regions tend to be subject to more frequent genomic rearrangements in malaria parasite, while the homology of the multi-gene families also reduces the accuracy of read mapping technologies. Our recent study found high coverage variability within chromosomal ends (Brashear et al., 2020), and upticks of coverage identified here toward chromosomal ends could be a result of read mismapping (Figure 1).

Despite seemingly close relationships (Table 2), proteins from multi-gene families were divergent enough from the reference that orthology-based analysis was unable to capture a large number of them (Figure 2), even though we were able to capture similar numbers of gene family members within each new *P. vivax* assembly using HMM profile searches. We were also capable of identifying large-scale structural variation around the *pir* genes and genes for exported proteins using CNV analyses, which suggests substantial genomic rearrangements around these gene families (Supplementary File 1). Interestingly, while NB45 is genetically differentiated from the other three genomes and harbors distinct deletions, it also shares some overlap of amplification with other samples, particularly LZCH1720 (Figures 4A, 5A, Table 4). Previous analyses have identified that some beneficial structural variations arise independently and are selected in parallel (Nair et al., 2008; Menard et al., 2013; Hostetler et al., 2016). Therefore, future analyses of large-scale CNVs within *P. vivax* could identify similarities. Another interesting observation was amplifications and deletions found in close proximity to one another on chromosome 9, suggesting the existence of chromosomal regions that are prone to genetic deletions or amplifications (Table 4).

*Pir* genes in particular are known for having a large degree of diversity and are divided into subfamilies (Lopez et al., 2013; Auburn et al., 2016; Chen et al., 2017). We analyzed the content of the *pir* gene family obtained through protein profile alignment, and found that subfamilies C and E were the largest gene family members in all assemblies and exhibit a degree of structure even within the subfamilies, which mirrors findings from previous analyses (Auburn et al., 2016; Chen et al., 2017). Interestingly, we found no members of PIR D aligned to the HMM profile for the *pir* gene family, and instead most proteins with high identity to the PIR D subfamily, under isolated searching, had higher similarity to the DUF3671-containing proteins with unknown functions (Table 3). Extensive comparisons of protein families in *P. vivax* has previously demonstrated that PIR subfamilies D, H and A are only loosely related to PIR proteins, with PIR subfamily D being the most dissimilar and having no homology blocks in common with other PIR subfamilies (Lopez et al., 2013). This study posited that PIR A, PIR H and especially PIR D should be considered separate gene families, which our data support. Motif comparisons demonstrated that PIR D subfamily members do not have common motifs with other PIR family members, and instead suggested strong homology with DUF3671. This would explain why previous studies have either represented no gene family members from PIR D, or have specifically used homology to the subfamily grouping to achieve representation (Auburn et al., 2016; Chen et al., 2017). Our data corroborates that PIR D proteins are dissimilar to other *pir* gene family members, and points to an alternative gene family united by the DUF3671 domain. PIR A and PIR H were not as disparate within our dataset and were identifiable by HMM profiling. However, previous phylogenetic analyses comparing various multi-gene families suggest common ancestry between PfEMP1, SicaVAR, and Surfin proteins, making it possible that new gene families could evolve via increasing divergence of existing subfamilies (Frech and Chen, 2013).

We found inter-sample variation within the *msp7*-B genes which would be difficult to detect through SNP profiling alone. Previous studies have shown the diversity of *msp*7 within species, finding that *msp7*-A paralogs are well conserved within species (Castillo et al., 2017), a finding well supported here (Figure 4). The differences in redundancy and interspecies conservation have previously been suggested to correspond to different roles in invasion for *msp7* subtypes A, B and C (Garzon-Ospina et al., 2016). Patterns of variation within the MSP7-B subfamily closely mimic whole genome findings, suggesting MSP7-B is a viable measure of population diversity (Figure 4). NB45 was differentiated from the other three samples in both whole genome analyses and analysis of *msp7* genes (Figure 4). Our previous results support that LZCH1720, LZCH1886 and LZCH1476 probably resulted from clonal expansion linked to a *P. vivax* outbreak in 2013, which would explain their higher relatedness as compared to NB45 (Brashear et al., 2020). The small sample size and close relationship among three of the four samples are also likely to result in the relatively low nucleotide diversity as compared to other previously surveyed *P. vivax* populations (Hupalo et al., 2016; de Oliveira et al., 2017).

We present here the first collection of high-quality *P. vivax* genome assemblies from the China-Myanmar border. This area is important in the scheme of malaria elimination due to the potential cross-border spread of malaria parasites (Lo et al., 2015, 2017). Previously, it has been demonstrated that the China-Myanmar border has substantial genetic separation from other areas in Southeast Asia. Further, *P. vivax* is the predominant form of malaria in the border region (Geng et al., 2019). Therefore, in-depth statistics on gene content and variability from *P. vivax* on the China-Myanmar border represent a benefit to the malaria community. Our genome assemblies presented here were able to provide further insight into the dynamics of gene families within *P. vivax* and constitute a new resource for malaria researchers.

## Data Availability Statement

The datasets generated for this study can be found in the NCBI database within bioproject PRJNA603279 (www.ncbi.nlm.nih.gov/bioproject/?term = PRJNA603279).

## Ethics Statement

The studies involving human participants were reviewed and approved by Bureau of Health of Kachin and the Institutional Review Board of Pennsylvania State University. Written informed consent to participate in this study was provided by the participants’ legal guardian/next of kin.

## Author Contributions

AB, LC, JG, AH, and JM contributed to study design and conceptualization. QF, YH, YL, YZ, and YC collected blood samples and extracted DNA. AB and ZW prepared genomic sequences. AB prepared assemblies and peptide analysis. AH and LD performed CNV analyses. AB wrote the first draft of the manuscript. All authors contributed to editing, revising, and approving manuscript.

## Conflict of Interest

The authors declare that the research was conducted in the absence of any commercial or financial relationships that could be construed as a potential conflict of interest.

## Footnotes

1 www.plasmodb.org
