# Supplementary material for: New Plasmodium vivax Genomes From the China-Myanmar Border
Source: Front Microbiol. 2020 Aug 11;11:1930. doi: 10.3389/fmicb.2020.01930 (PMC7432439; doi:10.3389/fmicb.2020.01930)
Supplement: Supplementary file 2 [file Data_Sheet_2.PDF]

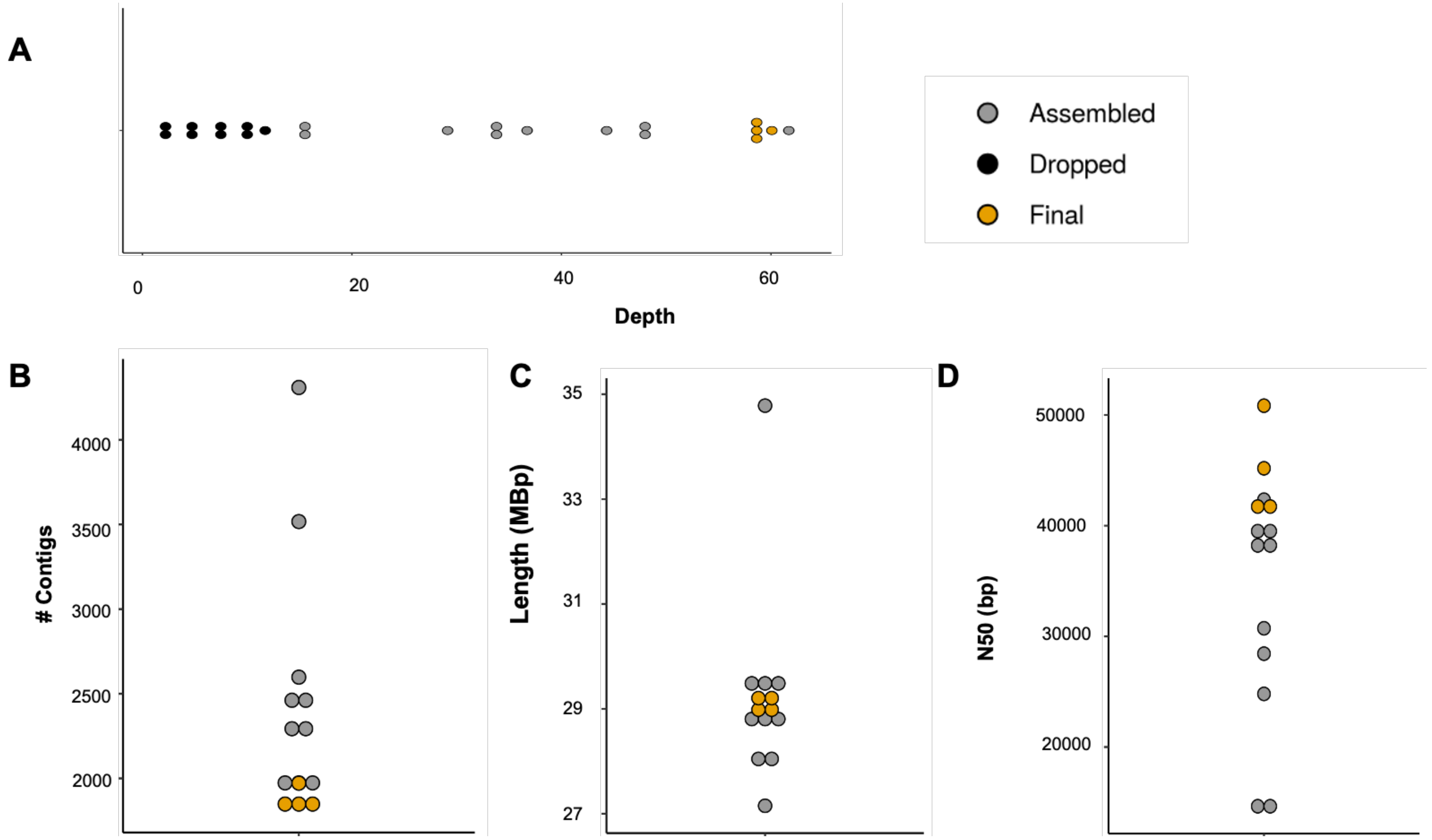

S1 Figure. Outcome of genome assembly for 13 field samples. Yellow dots represent those chosen for post-assembly correction and subsequent analysis. Black dots represent samples which were never assembled due to low coverage.

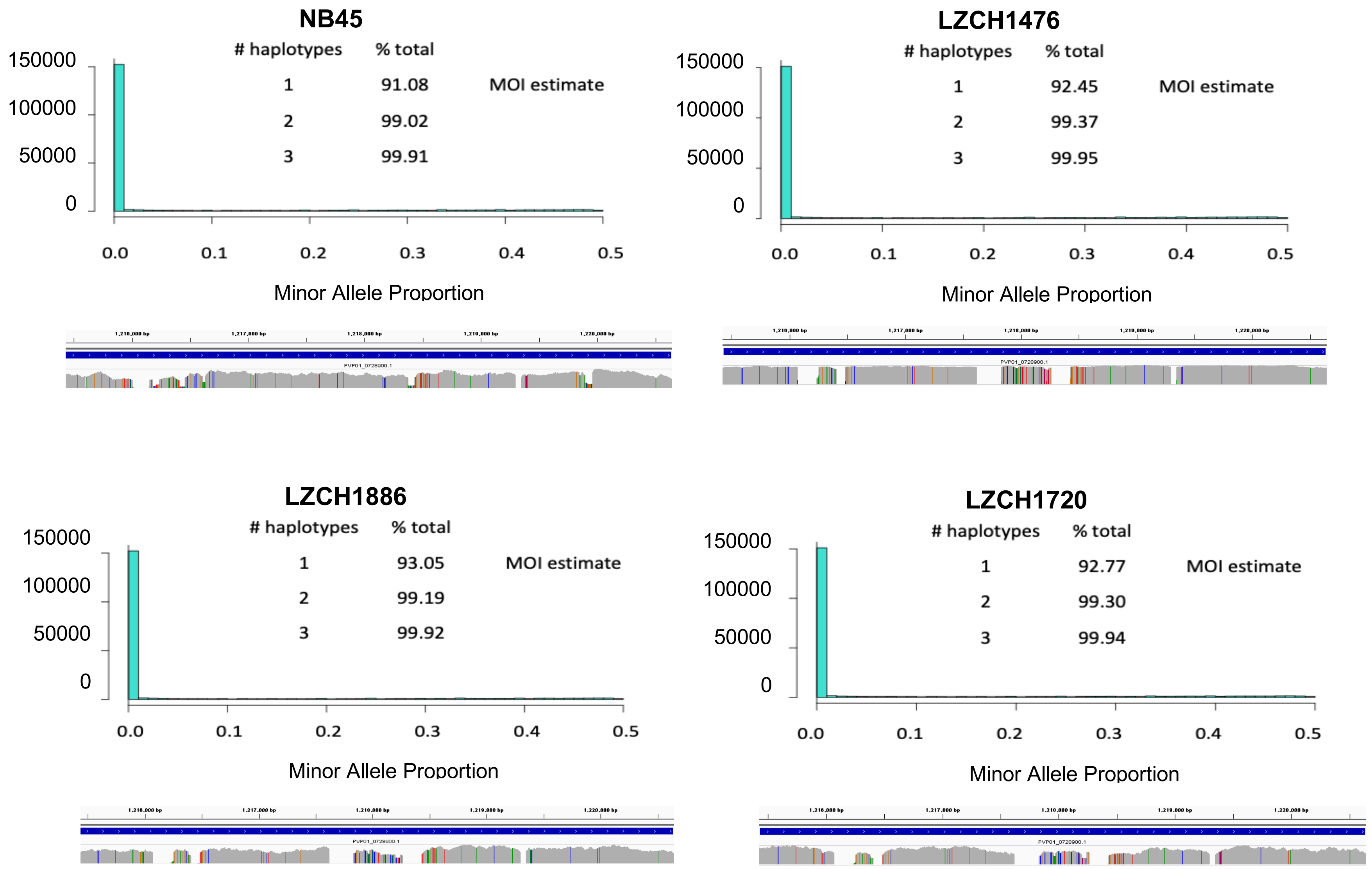

Supplementary Figure S2. Clonal confirmation for each of 4 high quality assemblies. For each sample, the top panel is minor allele proportion, overlaid with EST-MOI output statistics. The bottom panel is variants captured from the highly variable MSP1 gene.

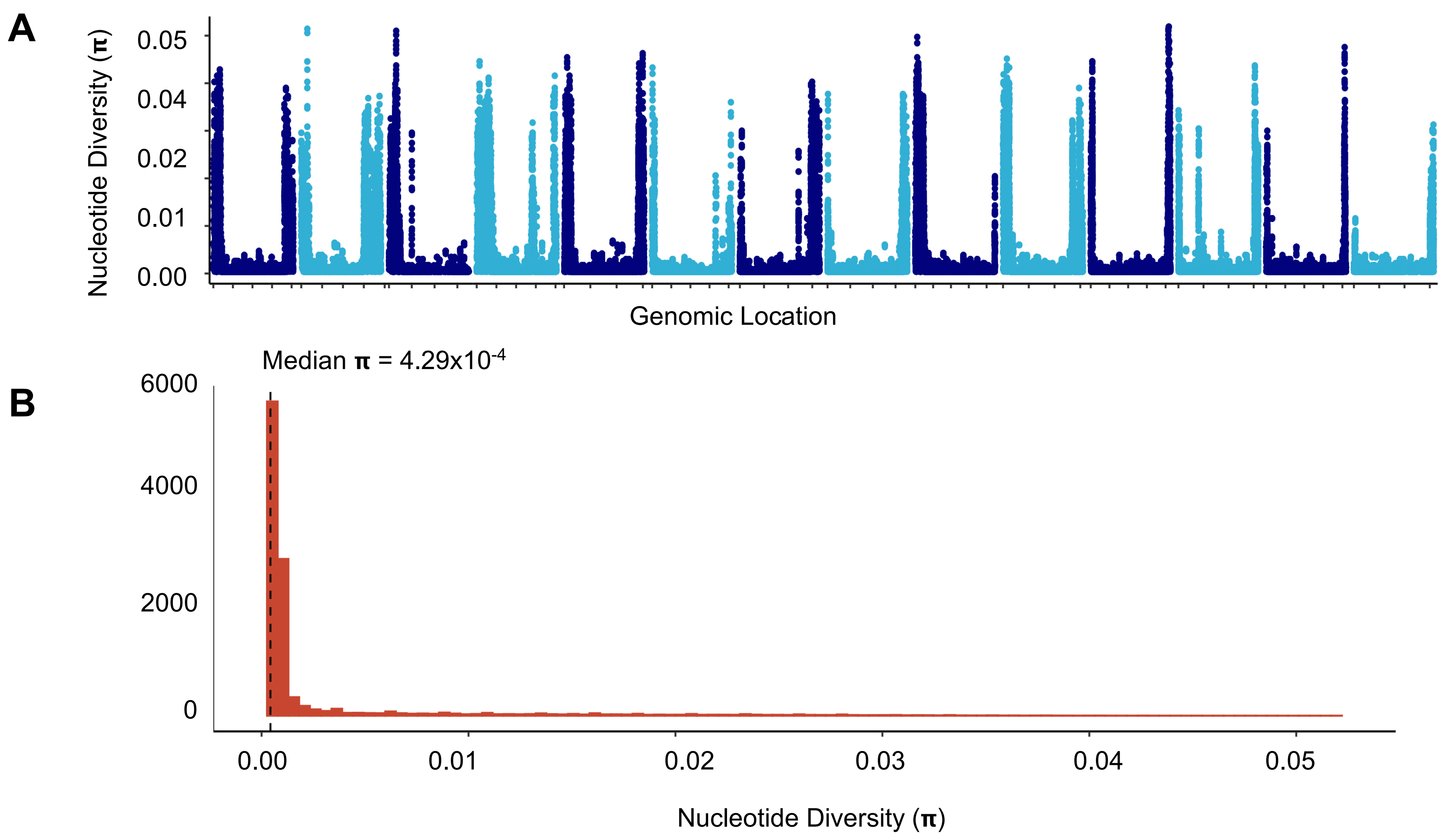

Supplemental Figure S3. Nucleotide diversity within *P. vivax* assemblies (**A)** Nucleotide diversity across each 1kb window (with 100 bp steps) across the genome **B)** Histogram showing the distribution of nucleotide diversity for genomic windows with the median labelled with a dashed line.

Supplemental Table S1. Chromosome sizes for each assembly, Sal1 and P01

Chromosome	P01	Sal1	NB45	LZCH1720	LZCH1886	LZCH1476	Assembly Average	Difference (P01)
**1**	1021664	830022	1053963	1112686	1400511	1140015	967539	-54125
**2**	956327	755035	988766	892613	964883	1001601	953137	-3191
**3**	896704	1011127	925387	918406	915105	907533	916608	19904
**4**	1012024	876652	1072677	1144657	1313989	1466271	1003140	-8885
**5**	1524814	1370936	1585137	1491934	1481498	1473037	1473388	-51427
**6**	1042791	1033388	1061655	1049589	1074209	1061251	1051857	9066
**7**	1652210	1497819	1801589	1619496	1780500	1633238	1598517	-53693
**8**	1761288	1678596	1743222	1920463	1750060	1783114	1764030	2742
**9**	2237066	1923364	2176055	2120076	2329902	2202669	2113658	-123409
**10**	1548844	1419739	1659084	1565681	1666072	1735928	1547153	-1691
**11**	2131221	2067354	2176200	2125501	2152277	2176476	2157614	26393
**12**	3182763	3004884	3205352	3309405	3157281	3232568	3136958	-45806
**13**	2093556	2031768	2390701	2430112	2427101	2423938	2091119	-2437
**14**	3153402	3120417	3168735	3230630	3195458	3300598	3211375	57973

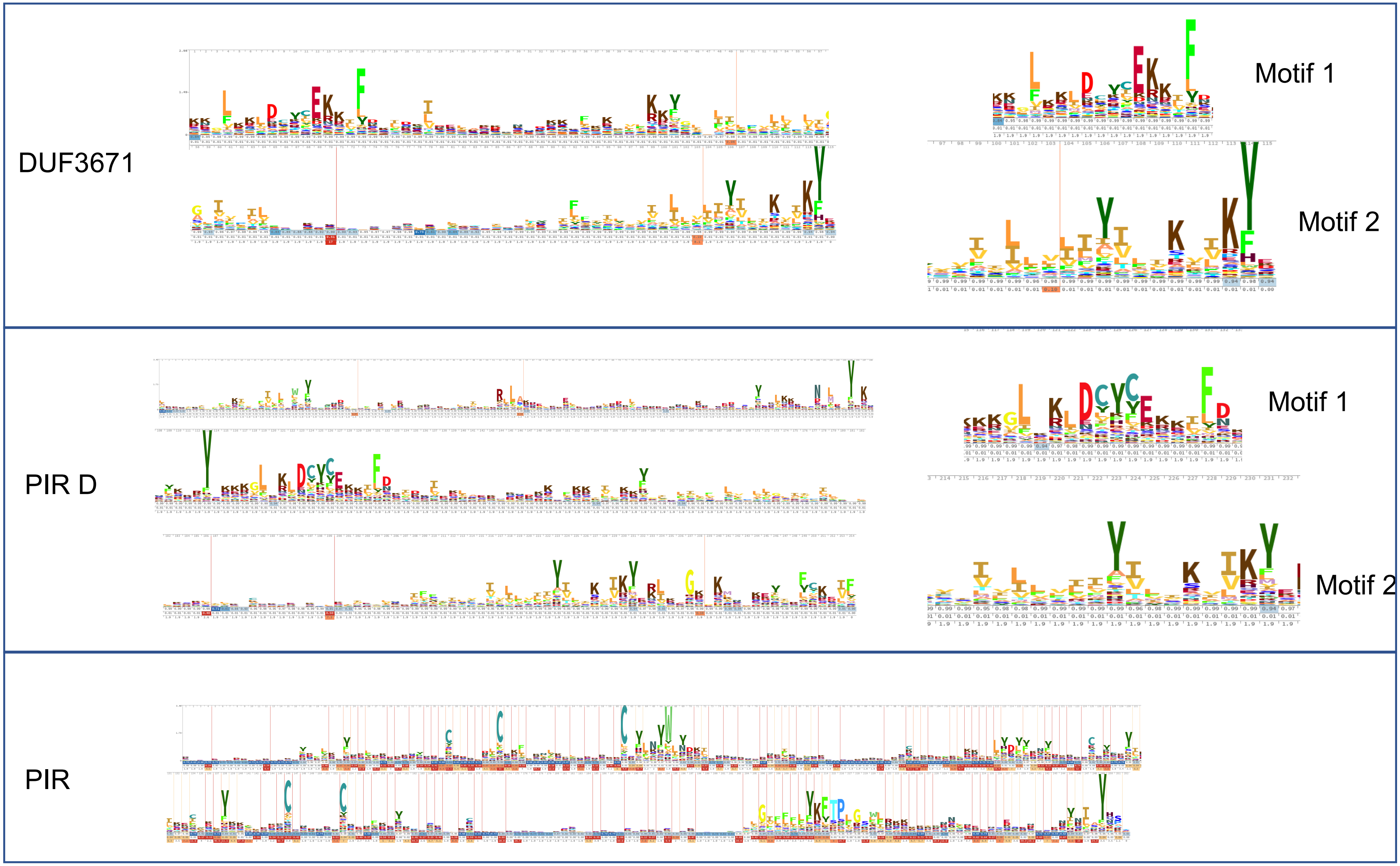

Supplemental Figure S4. HMM profile motifs for genes in the DUF3671 gene family, the PIR D characterized gene subfamily and the PIR gene family as a whole. Shared Motifs 1 & 2 are extracted on the right for DUF 3671 and PIR D families, while the left has the entire HMM profile skylign logo

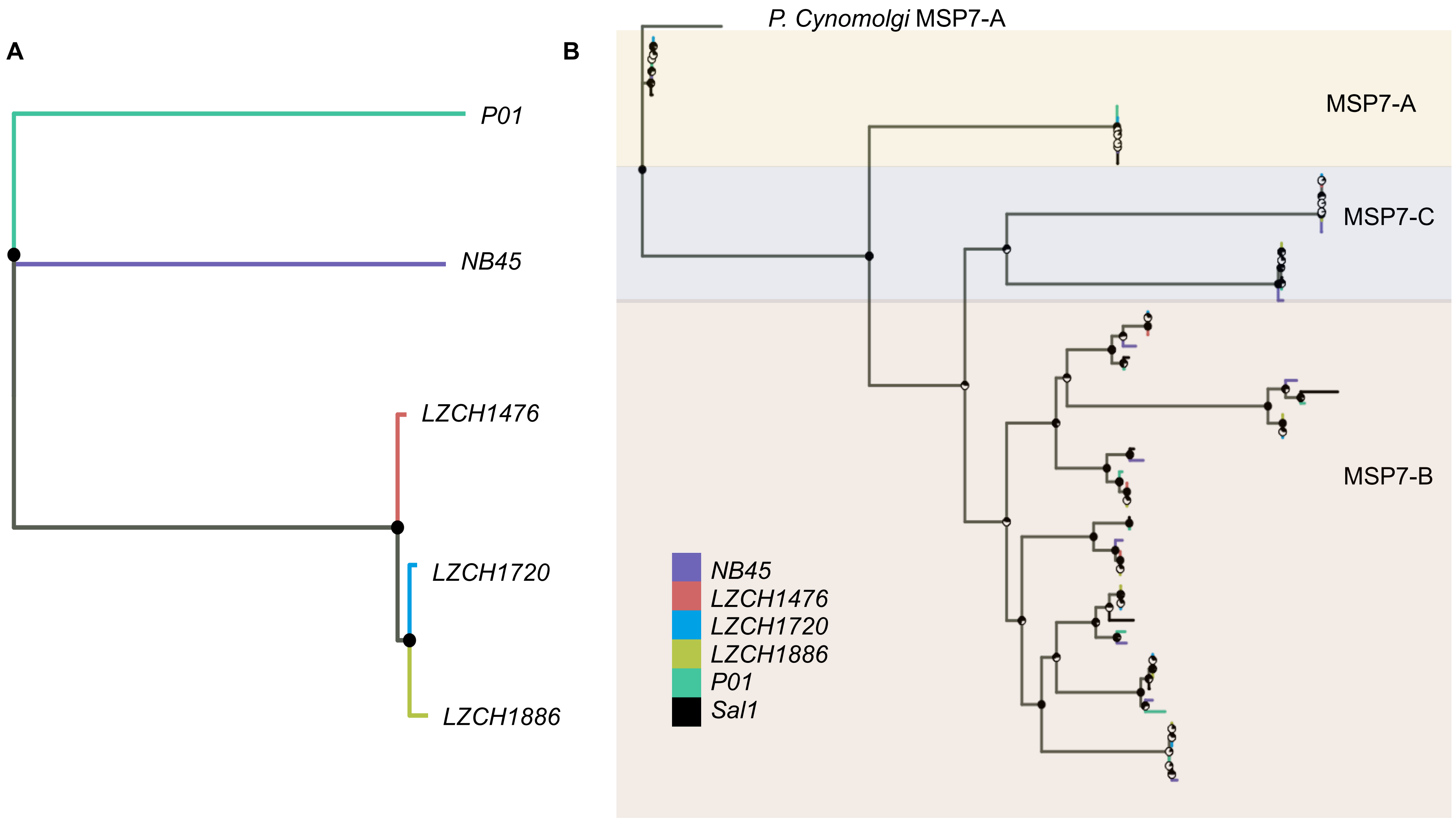

Supplemental Figure S5. Genetic diversity within *P. vivax* assemblies including bootstrap support from a maximum of 1000 bootstrap replicates. (**A)** Genetic diversity within the entire genome of the 4 assemblies compared to P01 based on SNPs from alignment to Sal1**.** Convergence was reached after 50 replicates. **B)** Diversity within MSP7 genes between different assemblies, Sal1 and P01. Pcym MSP7-A was used as an outgroup. Bootstrap values are represented by pie charts wherein completely black-filled circles have a bootstrap value of 100 and completely white circles have a bootstrap value of 0.
